# MRI guided copper deprivator activated immune responses and suppressed angiogenesis for enhanced antitumor immunotherapy

**DOI:** 10.7150/thno.102556

**Published:** 2025-01-01

**Authors:** Yinfeng Wang, Peng Wang, Huimin Li, Miao Yan, Feixue Ni, Li Zhang, Zhen Zhao, Wenjuan Gao, Guilong Zhang

**Affiliations:** 1 School of Pharmacy, Shandong Technology Innovation Center of Molecular Targeting and Intelligent Diagnosis and Treatment, Binzhou Medical University, Yantai 264003, China; 2 Department of Vascular Surgery, Shanghai Ninth People's Hospital, Shanghai Jiaotong University School of Medicine, Shanghai, China.; 3 Department of Urology, the First Affiliated Hospital of Anhui Medical University, Institute of Urology, Anhui Medical University and Anhui Province Key Laboratory of Genitourinary Diseases, Anhui Medical University, Hefei 230022, Anhui, China.

**Keywords:** Copper deprivation, Anti-angiogenesis, Antitumor immunity, MRI, Tumor metastasis inhibition

## Abstract

**Background:** Copper plays an important role in the regulation of PD-L1, suggesting that reducing copper levels within tumors may enhance anti-cancer immunotherapy.

**Methods:** Tumor microenvironment responsive copper nanodeprivator (TMECN) was developed for enhancing immunotherapy of tumor via the cross-link of mercaptopolyglycol bipyridine and dimercaptosuccinic acid modifying FePt nanoalloy using the disulfide bond.

**Results:** Upon entering tumor cells, the disulfide bond in TMECN is cleaved by the overexpressed glutathione, exposing abundance of sulfhydryl groups. Next, TMECN actively captured copper ions in the cancer cells, which triggered the self-assembly of TMECN. The reduced copper not only inhibited tumor neovascularization and PD-L1 transcription but also promoted the ubiquitination and degradation of PD-L1, blocking tumor immune escape. In addition, TMECN catalyzed Fenton reaction and produced reactive oxygen species (ROS) in cancer cells, inducing immunogenic cell death (ICD) of tumor. The inhibition of PD-L1 and the activation of ICD synergistically promoted cytotoxic T lymphocyte infiltration for tumor, evoked robust antitumor immune responses. In addition, the self-assembly of TMECN in tumor induced T_1_ to T_2_ switchable contrast imaging, which significantly improved accurate diagnosis of tumor.

**Conclusion:** TMECN could effectively inhibit tumor growth and metastases, meanwhile improve MRI contrast enhancement of tumor. The project will offer a simple strategy for enhancing MRI-guided antitumor immunotherapy.

## Introduction

Cancer immune evasion is considered to be a hallmark of tumor development 1. One mechanism that cancer cells protect themselves from the anti-tumor immune response by overexpressing programmed death ligand 1 (PD-L1) on cell membrane. The interaction of PD-L1 with the immune checkpoint protein programmed death receptor 1 (PD-1) expressed on lymphocytes activates immunosuppressive signals, leading to tumor immune escape and attenuating endogenous anti-tumor immune response 2,3. Starting with the approval of anti-cytotoxic T lymphocyte-associated antigen 4 (CTLA-4) and programmed death-1 (PD-1) gained by FDA, immune checkpoint inhibitors (ICIs) have made indelible achievements in cancer immunotherapy 4. However, despite the success of ICIs, only a minority of patients treated with ICIs achieve a durable response 5,6. Therefore, it was necessary to design and develop new approaches to improve the efficiency of tumor immunotherapy and reduce side effects.

Copper (Cu) is one of the essential trace elements in the body, which plays an important role in physiological processes including mitochondrial respiration, antioxidant reaction, and biological macromolecule synthesis 7,8. Because both copper deficiency and copper overload can cause damage to cells, copper homeostasis is strictly regulated. More and more research evidences show that Cu imbalance is related to a series of diseases, including Menkes disease, Wilson's disease, neurodegenerative diseases and cancer 9. Multiple investigations have demonstrated that tumor cells had more copper species than normal cells. These copper species can promote the migration and proliferation of endothelial cells between tumor sites as well as facilitate the production of angiogenic factors in tumor cells 10-12. Thus, copper chelation can systematically and significantly reduce copper concentrations and break cellular copper homeostasis, which suppresses copper transport, attenuates the proliferation and motility of endothelial and cancer cells, and inhibits tumor angiogenesis 13-16. In addition, the major copper ion channel transporter CTR-1 is positively correlated with the expression of PD-L1 in many cancers, but not in corresponding normal tissues 17. Moreover, copper depletion in cancer cells inhibits the phosphorylation of STAT3, epidermal growth factor receptor (EGFR), AKT and GSK3β. This inhibits PD-L1 transcription and promotes ubiquitination and degradation of PD-L1, thereby blocking immune escape. However, since copper is a coin-supporting factor for most enzymes or proteins, it involves in a wide range of metabolic processes 18-20. Therefore, developing a tumor-specific copper nanodeprivator will enhance precisely antitumor immunotherapy within low systemic toxicity.

**Scheme 1:**
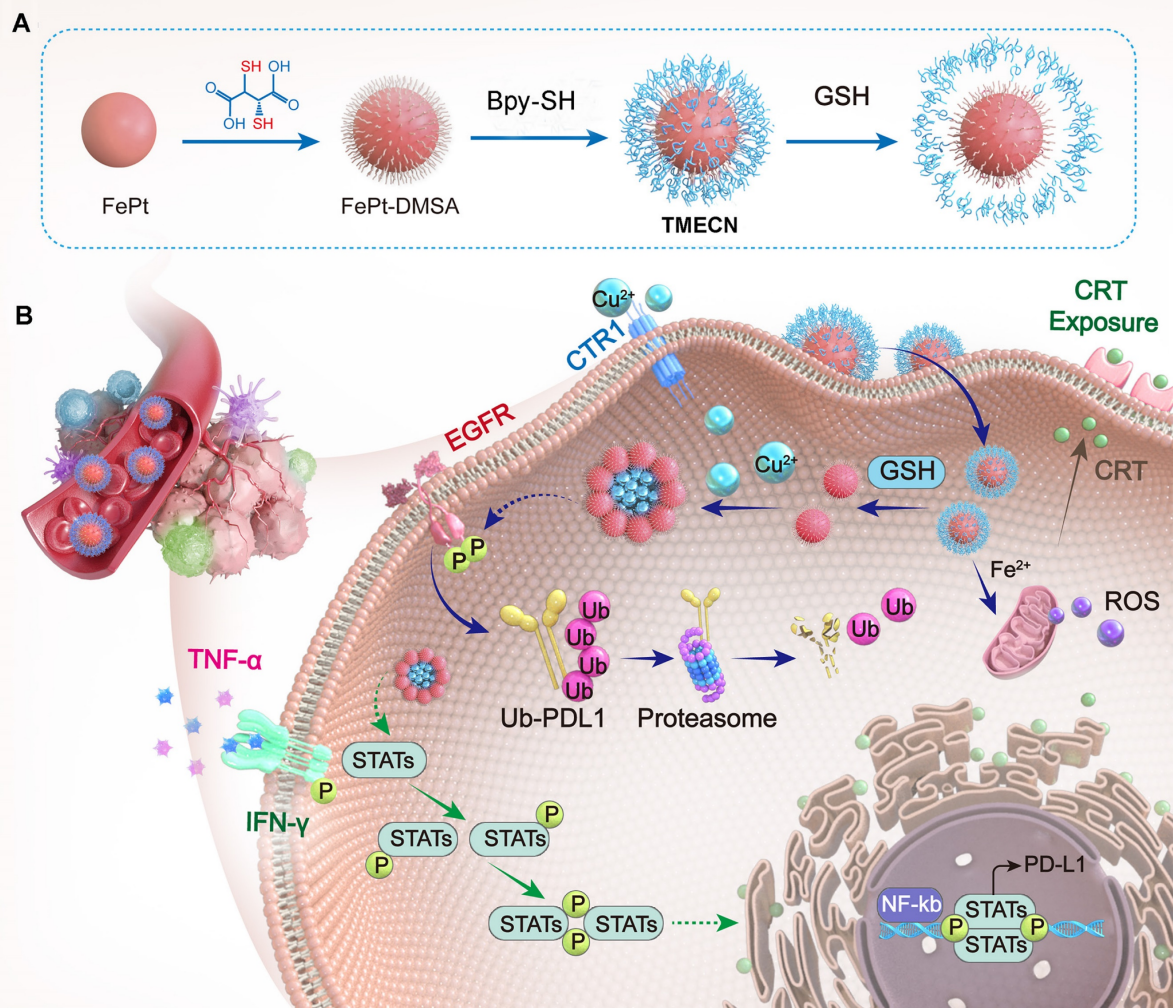
Schematic illustration of (A) TMECN preparation and (B) its anti-tumor mechanism.

In this work, we developed a tumor-microenvironment responsive copper nanodeprivator (TMECN) for enhancing tumor immune response and improving immunotherapy. As shown in **Scheme 1A**, the manufacturing process of TMECN is as follows: (1) Firstly, ultra-small FePt nanoalloys are synthesized using a modified high temperature thermal degradation method. (2) Then, FePt nanoalloys are modified by dimercaptosuccinic acid (DMSA) to achieve FePt-DMSA with abundance of thiol groups. (3) Finally, the thiol groups onto the surface of FePt-DMSA are protected via the cross-link of mercaptopolyglycol bipyridine (Bpy-PEG-SH) and FePt-DMSA, which fabricates TMECN. Upon entering tumor cells, the disulfide bond of TMECN is cleaved in response to the highly expressed GSH, exposing sulfhydryl groups on the surface of TMECN. Then, sulfhydryl groups on the surface of TMECN actively capture copper ions in cancer cells, inducing the self-assembly of TMECN and acquiring T_1_ to T_2_ switchable MRI contrast imaging. The reduced copper content not only inhibits tumor neovascularization, but also inhibits the phosphorylation of EGFR and STAT3, promotes ubiquitin-mediated degradation of PD-L1 and inhibits PD-L1 transcription, eventually blocks cancer immune escape (**Scheme 1B**). Moreover, the TMECN catalyze Fenton reaction to produce more reactive oxygen species (ROS), which elicits the immunogenic cell death (ICD) of tumor. Thus, this novel TMECN may inhibit tumor growth and metastasis, improving the survival rate of mice. This Cu removal strategy provides a great approach for enhancing MRI-guided antitumor immunotherapy.

## Results and Discussion

### Preparation and physico-chemical property characterization of TMECN

**Figure 1:**
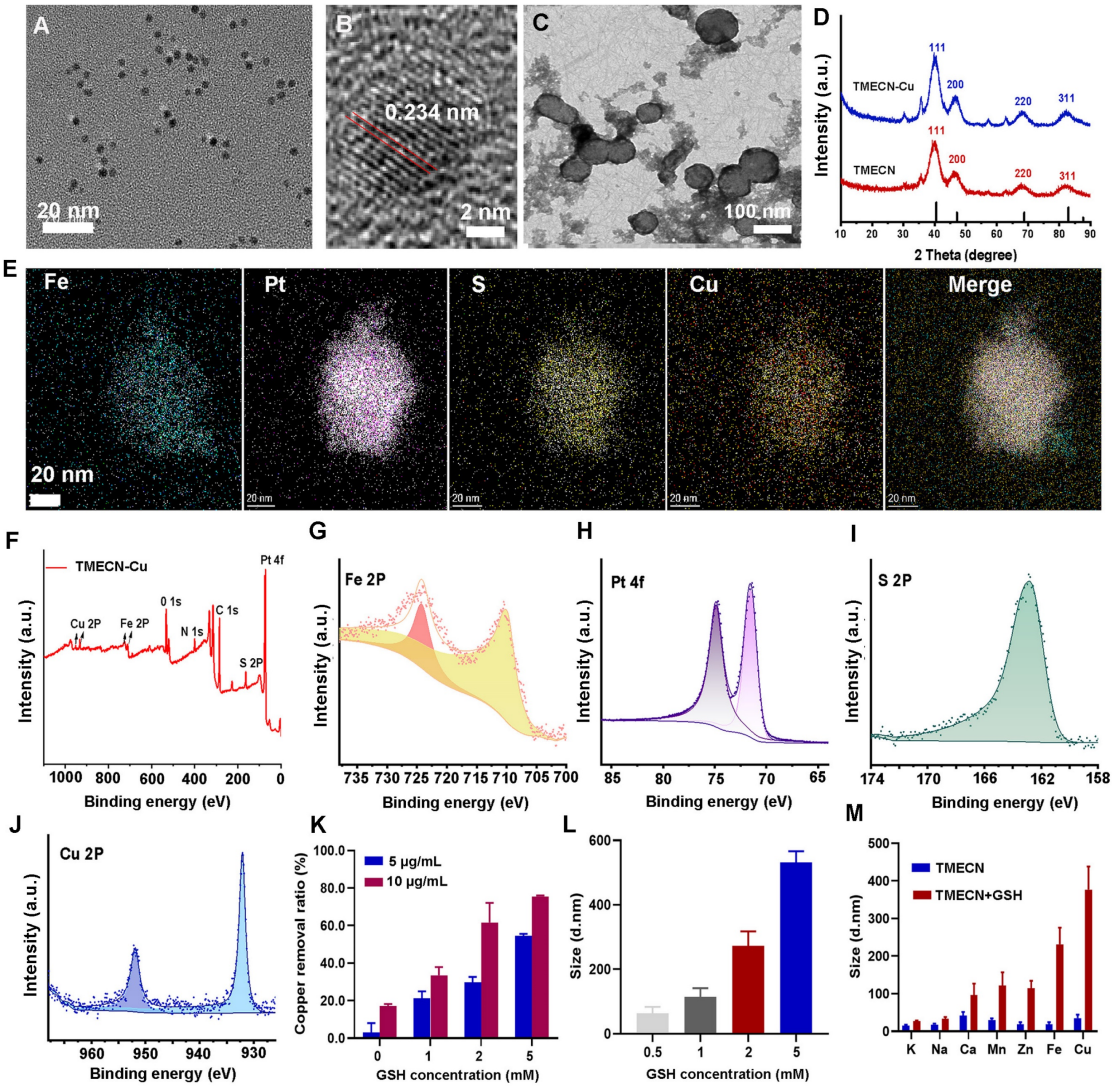
(A) TEM image and (B) high-resolution TEM image of TMECN. (C) TEM image of TMECN-Cu. (D) XRD spectra of TMECN and TEMCN-Cu. (E) Elemental mapping images of TMECN-Cu aggregates. (F) XPS full spectra, (G) Fe2p spectra, (H) Pt4f spectra, (I) S2p spectra, and (J) Cu2p of TMECN-Cu aggregates. (K) Copper removal efficiency of TMECN under different GSH concentrations. (L) Hydrodynamic particle size of TMECN-Cu after treatment with different concentrations of GSH. (M) Hydrodynamic particle size change of TMECN at different metal ions treatments in the absence and presence of GSH (5 mM).

Transmission electron microscopy (TEM) images showed that hydrophobic FePt nanoalloys had a uniform size with 2-4 nm (**Figure S1A**). Subsequently, the hydrophobic FePt nanoparticles were modified by dimercaptosuccinic acid (DMSA) to fabricate hydrophilic FePt-DMSA. As shown in **Figure S1B**, FePt-DMSA had a good monodispersity in the aqueous solution, implying the successful modification. To verify the ability of FePt-DMSA to capture Cu ions, the colloidal solution of FePt-DMSA was added into 60 µM of free Cu ions. Significant aggregates (FePt-Cu) were observed by TEM image after free Cu ions treatment, confirming copper-initiated the cross-linking of FePt-DMSA (**Figure S1C**). This result demonstrated that Cu ions could be effectively captured by FePt-DMSA and initiate the self-assembly of FePt nanoalloy. Subsequently, we further investigated the selectivity of FePt-DMSA to capture Cu ions via observing hydrodynamic size variation. Results showed that FePt-DMSA almost had no response to K^+^, Na^+^, Zn^2+^, and Mg^2+^ ions (**Figure S1D**), however it had significant response to Fe^3+^ and Cu^2+^. Moreover, FePt-DMSA had stronger chelation with Cu^2+^ ions compared to Fe^3+^ ions. Consideration that the intravenous administration might induce the inactivation of FePt-DMSA to capture Cu ion in tumor because of the presence of Fe^3+^ and Cu^2+^ ions in blood. In order to avoid this, Bpy-PEG-SH as a protective group was conjugated with FePt-DMSA via disulfide bond and then fabricated TMECN, which hindered the action of the sulfydryl goups and transition metal ions in blood. TMECN had similar morphology and dispersion with FePt-DMSA (**Figure 1A**). In addition, TMECN showed a narrow hydrodynamic size distribution of approximately 10 nm, which was larger than TEM observation due to the presence of surface hydration (**Figure S1E**). In addition, a significant lattice fringe with the interplanar spacing of 0.234 nm was observed in **Figure 1B**, corresponding to (111) plane of FePt nanoalloy. Zeta potential of TMECN was significantly increased compared to FePt-DMSA, demonstrating the successful fabrication of TMECN (**Figure S1F**). These findings indicate that TMECN as a copper nanodeprivator is successfully fabricated.

In order to assess the ability of copper removal in response to GSH, the colloidal solution of TMECN was incubated with free Cu^2+^ ions (60 μM), GSH (5 mM), and GSH (5 mM)+Cu^2+^ ions (60 μM) for 30 min and photographed for observation. The solution of TMECN treated with free Cu^2+^ ions or GSH had no significant variation, however that with GSH+Cu^2+^ ions obviously aggregated and even precipitated (**Figure S2**), indicating that the ability of TMECN to capture Cu^2+^ ions could be activated in the presence of GSH. Subsequently, the GSH+Cu^2+^ ions treated TMECN was observed by TEM, and the image showed abundance of assemblies (TMECN-Cu) (**Figure 1C**). Crystal structures of TMECN before and after capturing copper were characterized by X-ray diffraction (XRD) and X-ray photoelectron spectroscopy (XPS). Crystal structure of TMECN had no any variation before and after capturing copper (**Figure 1D**). Elemental mapping analysis and XPS full spectrum of TMECN-Cu showed the presence of Fe, Pt, S, and Cu elements (**Figure 1E and 1F**), moreover these elements were uniformly distributed in TMECN-Cu nanoassembly. These results demonstrated that TMECN could effectively capture Cu ions. Fe2p and Pt4f peaks of TMECN appeared at 723.88 eV, 710.18 eV, 74.88 eV, and 71.58 eV, respectively (**Figure 1G-J**), indicating the presence of Fe (II) and Pt (0). In addition, S2p peaks can be seen at 162.88 eV, corresponding to the binding energy of the S-Cu bond. Cu2p peaks were also observed at 932.08 eV and 951.88 eV, suggesting the presence of Cu (II). Results proved that TMECN was quickly activated by GSH, actively captured Cu^2+^ ions, and then formed TMECN-Cu nanoassembly.

Based on this, we further investigated the effects of TMECN concentrations on copper removal efficiency. The solution of Cu^2+^ ions (60 µM) were treated with TMECN in the presence of different concentrations of GSH for 12 h. As shown in **Figure 1K**, copper removal increased with increasing concentrations of TMECN and GSH. Copper clearance was up to 76.33% at TMECN (10 μg/mL) and GSH (5 mM). In addition, **Figure 1M** showed the GSH concentration-dependent aggregation behavior of TMECN mediated copper clearance. Moreover, the hydrodynamic size of TMECN-Cu nanoassembly sharply increased from 10 nm to over 100 nm even if GSH at 1 mM **(Figure 1L)**, indicating that TMECN was very sensitive to GSH. In addition, TMECN almost had no any response to metal ions in the absence of GSH. However, TMECN had the strongest selectivity for Cu ions in the presence of GSH (**Figure 1N**). In addition, the morphology changes of TMECN were observed by TEM images after treatment with Cu^2+^ ions or Cu^2+^ ions plus different concentrations of GSH (0.5, 1, 2, 5 mM). As shown in **Figure S3**, the morphology of TMECN treated with free Cu^2+^ ions had no significant variation. However, the addition of GSH obviously induced the aggregation of TMECN, moreover the aggregates gradually increased with the increase of GSH concentrations, consistent with the results of hydrodynamic size variation. The above study proved that we successfully prepared an effective GSH-responsive copper nanodeprivator. We also explored the ability of TMECN to catalyze Fenton reaction and produce ·OH by determining 3,3',5,5'-tetramethylbenzidine (TMB) color assay. As shown in **Figure S4A-B**, the production of ·OH depended on both pH and TMECN concentration, confirming that TMECN catalyzed Fenton reaction and produced ROS. In addition, to further validate copper removal-mediated antitumor effect, the dopamine hydrochloride modified FePt nanoalloy (FePt-Dopa) was set as control group, and it cannot capture Cu^2+^ ions. The morphology and size of FePt-Dopa had no significant difference compared to TMECN (**Figure S5A-B**). Zeta potential of FePt-Dopa was 30.47 mV (**Figure S5C**), and the nanoparticles still remained monodisperse without aggregation after incubating with Cu ions (**Figure S5D**). This result indicated that FePt-Dopa has the similar physicochemical property with TMECN, but it wasn't able to respond for free Cu^2+^.

### Cellular uptake, ROS production, cytotoxicity, and anti-tumor immune mechanisms *in vitro*

**Figure 2:**
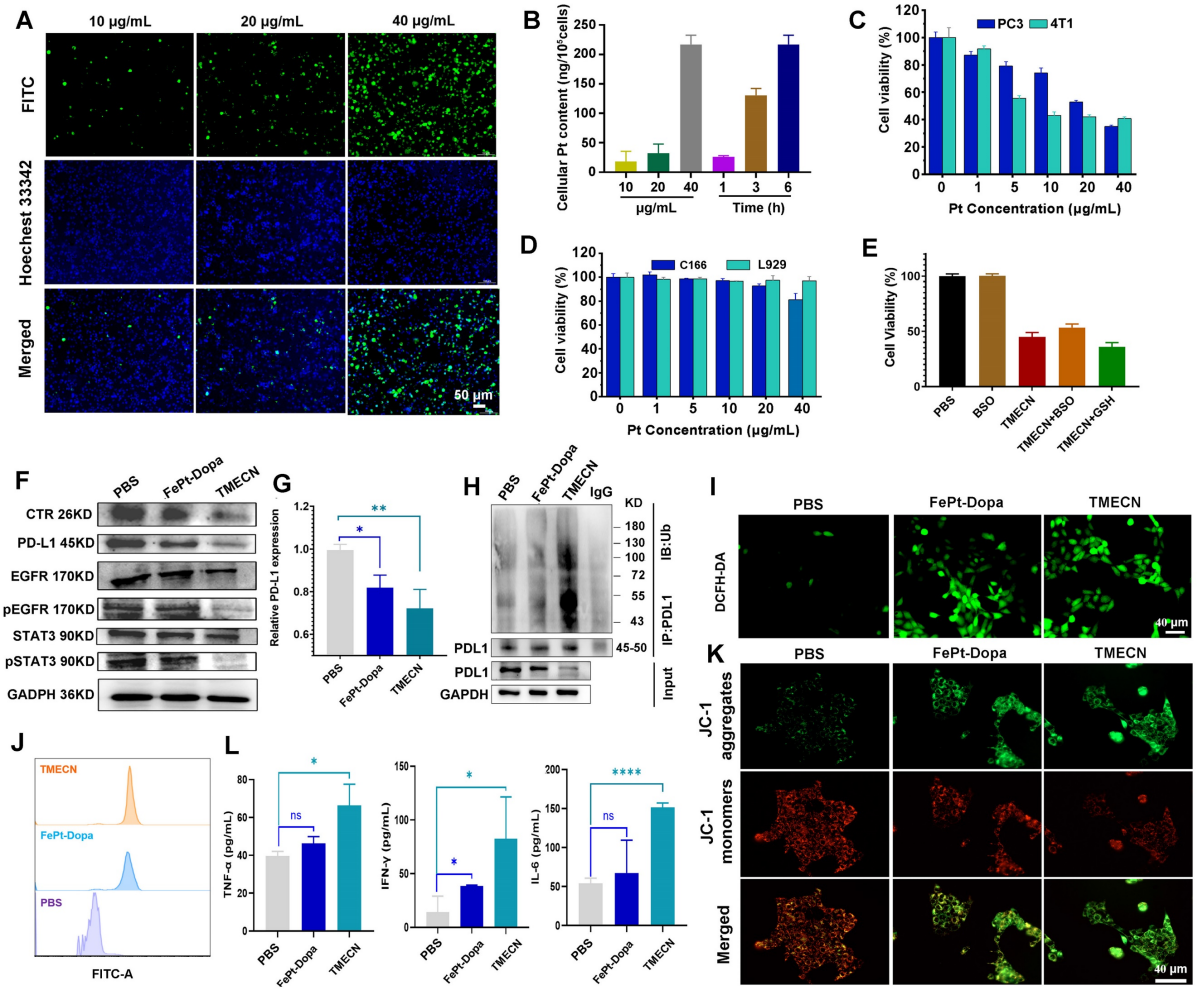
The internalization process of 4T1 cells: (A) CLSM observation after different concentrations of TMECN treatment. Scale bar: 50 μm. (B) ICP-MS analysis after different treatments. (C) The viability of 4T1 cells and PC-3 cells treated with different concentrations of TEMCN. (D) The viability of C166 and L929 cells treated with different concentrations of TEMCN. (E) The viability of 4T1 cells treated with different samples. (F) Western blot analysis of the protein expression of PD-L1, CTR, EGFR, p-EGFR, STAT3, p-STAT3 after different treatments. (G) Real-time qPCR analysis of PD-L1 mRNA in 4T1 cells after different treatments. (H) Co-immunoprecipitation analysis for ubiquitination process of PD-L1 after different treatments. (I) CLSM observation and (J) flow cytometry analysis for ROS production of 4T1 cells treated with different samples. (K) CLSM observation for mitochondrial membrane potential using JC-1 staining. (L) The production of TNF-α, INF-γ and IL-6 in 4T1 cells detected by ELISA. Data were expressed as mean ± SD, n = 3, *p < 0.05, **p < 0.01, ***p < 0.001, ****p < 0.0001, ns, not significant.

We observed the internalization of TMECN by confocal laser microscopy (CLSM), and TMECN was labeled with FITC to indicate the location of nanoparticles in tumor cells. As shown in **Figure 2A**, the green fluorescence of 4T1 cells gradually strengthened with increased TMECN concentration. ICP-MS results quantitatively confirmed that the uptake of TEMCN was time- and dose-dependent (**Figure 2B**). Encouraged by these results, we further evaluated the cytotoxicity of TMECN on cancer cells and normal cells by MTT experiments. As shown in **Figure 2C-D**, TMECN significantly inhibited the viability of 4T1 and PC3 in a concentration-dependent manner. However, the TMECN exhibited negligible toxicity to C166 and L929 cells, indicating that TMECN was low toxicity to normal cells. These results demonstrated that cytotoxicity of TMECN had excellent tumor specificity. To elucidate the mechanism of cell death induced by TMECN, we hypothesized that the GSH-initiated Cu capture might be a key factor for inducing cancer cell death. Subsequently, the cytotoxicity of TMECN on 4T1 cells was further detected under the conditions of GSH inhibitor (BSO) and additional supplementation of GSH (**Figure 2E**). Notably, TMECN+GSH showed stronger toxicity on 4T1 cells than the TMECN. While, when BSO were added, the viability of TMECN-treated cells showed a degree of recovery. This finding implies that TMECN mediated copper deficiency in cancer cells might effectively inhibit tumor cell proliferation.

Copper removal blocked EGFR and STAT3 signaling pathways, inhibiting PD-L1 gene transcription and promoting the ubiquitylation degradation of PD-L1 protein. Therefore, we examined the expression level of PD-L1 and its related signaling pathway under different treatments. **Figure 2F and Figure S6** displayed that the phosphorylation of EGFR and STAT3 were down-regulated after treatment with TMECN. The expression of CTR-1 was dramatically decreased in TMECN treated group compared to the PBS and FePt-Dopa groups. Similarly, the expression of PD-L1 was dramatically down-regulated. Moreover, RT-qPCR results further confirmed the down-regulation of PD-L1 expression (**Figure 2G**), indicating that TMECN effectively disrupted PD-L1 transcription. Meanwhile, the co-immunoprecipitation assay also showed a significant decrease on PD-L1 expression after TMECN treatment (**Figure 2H**), confirming ubiquitination degradation process. The results demonstrated that TMECN treatment reduced PD-L1 expression through two modes of action: (1) at the transcriptional level by down-regulating the STAT3 signaling pathway and inhibiting the transcription of PD-L1; (2) at the post-translational level by inhibiting the phosphorylation of EGFR, which promotes the ubiquitination and degradation of PD-L1.

We further investigate the ability of TMECN to generate ROS in 4T1 cells using an 2,7-dichlorodihydrofluorescein diacetate (DCFH-DA) probe. Results showed that the FePt-Dopa or TMECN induced substantial ROS generation (**Figure 2I-J**). Importantly, excessive ROS production damaged the mitochondria, resulting in the reduction of mitochondrial membrane potential (**Figure 2K**). The GSH levels of 4T1 cells treated with PBS, FePt-Dopa, and TMECN were further analyzed via CLSM observation and flow cytometry. Then, the GSH level in tumor cells after different treatments was detected by GSH probe (monochlorobimane, MCB). Dil was used to label cell membrane. As shown in **Figure S7A**, CLSM images showed that the GSH fluorescence of 4T1 cells treated with TMECN was significantly lower than that with FePt-Dopa and PBS, verifying TMECN-mediated GSH depletion. The results of flow cytometry were consistent with the CLSM observation (**Figure S7B**). In addition, ROS damaged the mitochondria, resulting in immunogenic cell death (ICD) and then releasing cytokines. Subsequently, high mobility group protein B1 (HMGB1) release and calreticulin (CRT) exposure were examined by immunofluorescence staining at cellular level. 4T1 cells were treated with PBS, FePt-Dopa, and TMECN (Pt: 10 μg mL^-1^), respectively. The results showed that the intranuclear fluorescence of HMGB1 in TMECN group was lowest compared to other groups (**Figure S8A**). In addition, compared to other groups, TMECN group showed the strongest CRT exposure. These results demonstrated that TMECN-mediated ROS production significantly induced ICD effect of tumor cells (**Figure S8B**). After that, we further confirmed whether TMECN-induced ICD effect could promote the maturation of DCs. Mouse dendritic cells (DC2.4) were treated with PBS, FePt-Dopa, and TMECN for 24 h, respectively. The proportion of CD80^+^ CD86^+^ DCs in TMECN group was significantly higher than that in PBS and FePt-Dopa groups, implying that TEMECN-induced ICD promoted DC maturation (**Figure S8C**). We also evaluated the secretion of cytokines (IL-6, TNF-α, and IFN-γ) in the supernatant of tumor cells by ELISA kit. Elevated levels of TNF-α, IFN-γ and IL-6 were observed in the supernatants of TMECN-treated 4T1 cells (**Figure 2L**). The above results preliminarily proved that TMECN could inhibit the expression of PD-L1 and induce ROS production in tumor cells, thus stimulating a strong immune response.

### *In vitro* antiangiogenic effects of TMECN

**Figure 3:**
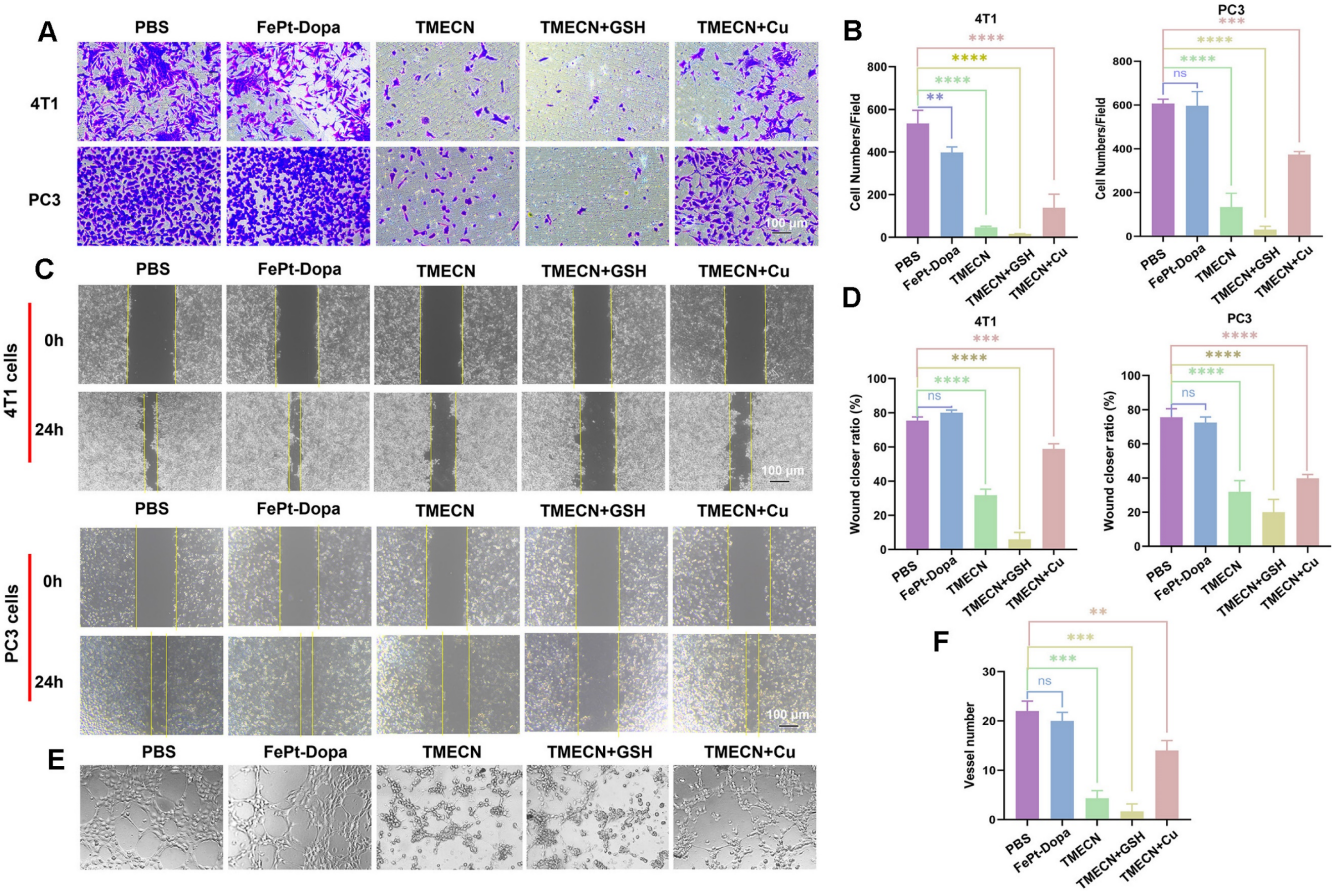
(A, B) Invision and (C, D) migration of 4T1 and PC3 cells treated with different samples. (E, F) Tube formation abilities of C166 cells treated with different samples. Data were expressed as mean ± SD, n = 3, *p < 0.05, **p < 0.01, ***p < 0.001, ****p < 0.0001, ns, not significant.

Copper promotes neovascularization by facilitating endothelial cell migration and invasion 21. Considering that cell migration and invasion are two key factors affecting tumor angiogenesis, we evaluated the effects of TMECN on tumor cell migration and invasion by transwell and wound healing assays. Compared with PBS group, FePt-Dopa almost didn't inhibit the invasion and migration of 4T1 cells and PC3 cells (**Figure 3A-D**). However, the invasion and migration of 4T1 and PC3 cells treated with TMECN were significantly inhibited, suggesting that TMECN induced copper deficiency could significantly affect the motility of 4T1 and PC3 cells. In addition, the inhibition of 4T1 and PC3 cells on invasion and migration was further strengthened after additional supplementation of GSH (5 mM), moreover the addition of free Cu^2+^ significantly recovered the migration and invasion of 4T1 and PC3 cells, further confirming that Cu element in tumor cells played an important role for the invasion and migration. Next, we further evaluated the effect of TMECN on angiogenesis ability of C166 cells using tube formation experiments (**Figure 3E-F**). In the PBS and FePt-Dopa treated groups, C166 cells formed a distinct tube network on the matrix gel-coated plates, indicating that FePt-Dopa had no any action on anti-angiogenesis. Remarkably, most of the tubes remained open and an increase in the number of dissociated cells was clearly observed in TMECN group, indicating that TMECN severely impaired C166 cell's tube-forming ability. Moreover, the supplementation of GSH further strengthened this trend. Nevertheless, when TMECN was pretreated with GSH and free Cu^2+^ ions, the number of tube formation in C166 cells treated with TMECN significantly increased. These results suggested that TMECN with strong GSH-responsive copper elimination ability have potent anti-angiogenesis effect.

### Anti-tumor activity investigation of TMECN* in vivo*

**Figure 4:**
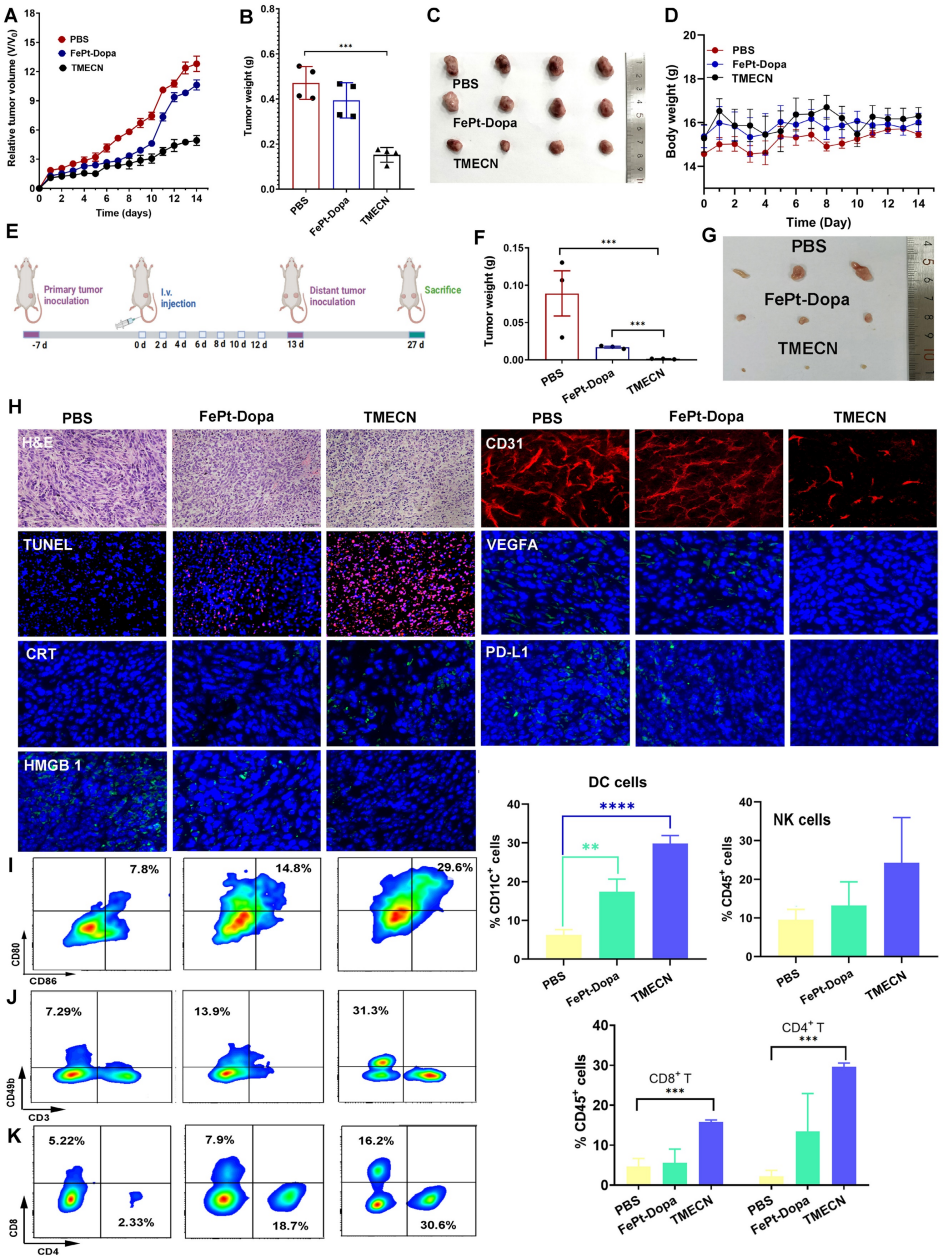
Anticancer effect of 4T1 tumor-bearing mice after different treatments. (A) Tumor volumes change, (B) tumor weights, (C) corresponding tumor photos, and (D) body weight change of tumor-bearing mice treated with different treatments. (E) Implementation plan for bilateral tumor experiments. (F) Corresponding distal tumor weights and (G) distal tumor photo of mice treated with different samples. (H) H&E, TUNEL, CD31 whole-mount staining, and expression of CRT, HMGB1, PD-L1, and VEGFA by IF in tumor tissues after different treatments. (I-K) Flow cytometry analysis for immune cell infiltration in tumors including CD8^+^ T cells, CD4^+^ T cells, and NK cells, as well as the maturation of DCs. Data were presented as mean ± SD, n = 3, *p < 0.05, **p < 0.01, ***p < 0.001, ****p < 0.0001, ns, not significant.

Encouraged by the *in vitro* results, we further explored the anticancer potential of TMECN* in vivo*. Firstly, the 4T1-bearing mice subjected to equivalent dose of FePt-Dopa were used as a control and PBS groups were set as the negative control. As shown in **Figure 4A**, tumor volume of mice treated with FePt-Dopa was slightly lower than Saline group, which might be originated from the anticancer effect of FePt-mediated ROS production. Notably, tumor volume of TMECN-treated mice was significantly smallest than that with PBS and FePt-Dopa, indicating that TMECN had outstanding antitumor activity. In addition, **Figure 4B-C** showed the smallest tumor size and lightest tumor weight in TMECN group when compared to Saline and FePt-Dopa group, further confirming excellent anticancer activity. There was no significant change in body weight during treatment process, suggesting the negligible systemic toxicity of TMECN (**Figure 4D**). To study copper stripping-mediated anti-tumor immune responses, we constructed a bilateral tumor mouse model (**Figure 4E**). After 7 doses, the primary tumors of mice were excised for immunoassay. Next, the mice were rechallenged by 4T1 cells in their right side at the 13th day after inoculation without any further treatment. Finally, the distant tumors were excised from the sacrificed mice on day 27, photographed, and then weighed. As shown in **Figure 4F-G**, TMECN group showed the smallest tumor size and lightest tumor weight compared to PBS and Fe-Pt-Dopa groups, suggesting that TMECN induced durable anti-tumor immune responses and then inhibited distant tumor growth.

Hematoxylin and eosin (H&E) staining results showed that TMECN treatment induced more apoptotic cells and numerous necrotic areas compared to PBS and FePt-Dopa treatment (**Figure 4H**). In accordance with the tumor growth results, we found that the apoptosis degree of TMECN treated tumor sections was also significantly higher than that of the other two groups by TUNEL staining assay. CD31 whole-mount staining showed that TMECN-treated-tumors had the least vascular distribution. In addition, TMECN-treated tumor also showed the lowest expression level of VEGFA. These results demonstrated that TMECN had strong ability to inhibit tumor angiogenesis *in vivo*. Then, copper levels in the tumor were detected by ICP-MS. TMECN significantly reduced copper levels in tumors, confirming that a GSH concentration-dependent copper-removal manner significantly inhibited tumor angiogenesis (**Figure S9**). As previous report, excessive ROS can induce immunogenic cell death (ICD) 22,23, resulting in the release of calreticulin (CRT) and high mobility group protein B1 (HMGB1) 24-26. CRT serves as an "eat me" signal that promotes phagocytic uptake of apoptotic cells 27, and HMGB1 is a highly conserved nuclear protein which passively released by dying, stressed or injured cells 28. **Figure 4H** showed that the fluorescence of CRT significantly increased on cell membrane of TMECN group, compared with that of PBS and FePt-Dopa groups. The intranuclear fluorescence intensity of HMGB1 in TMECN group was much weaker than that in PBS or FePt-Dopa group. These results demonstrated that TMECN effectively induced ICD, which will promote DC maturation and cytotoxic T lymphocyte (CTL) infiltration for tumor. Notably, TMECN-treated-group presented much lower PD-L1 expression, further confirming Cu removal-mediated PD-L1 downregulation. These results were consistent with the* in vitro* results. To further confirm our hypothesis that TMECN could evoke robust antitumor immune responses, the primary tumor was collected and dissociated into a single cell suspension to analyze the proportion of tumor-infiltrating dendritic cells (DCs), NK cells, CD8^+^ T cells, and CD4^+^ T cells. As shown in **Figure 4I-K**, TMECN treatment significantly promoted DCs maturation and increased the number of NK cells. As expected, cytotoxic T lymphocytes (CD8^+^ T cells) and helper T lymphocytes (CD4^+^ T cells) infiltration in tumor tissues also increased significantly in TMECN group. These results suggested that TMECN triggered systemic anti-tumor responses by promoting DC maturation, NK cell proliferation, and facilitating the infiltration of CTL, indicating a potential role of TMECN in shaping tumor immune microenvironment.

### TMECN-mediated anti-tumor metastasis* in vivo.*

**Figure 5:**
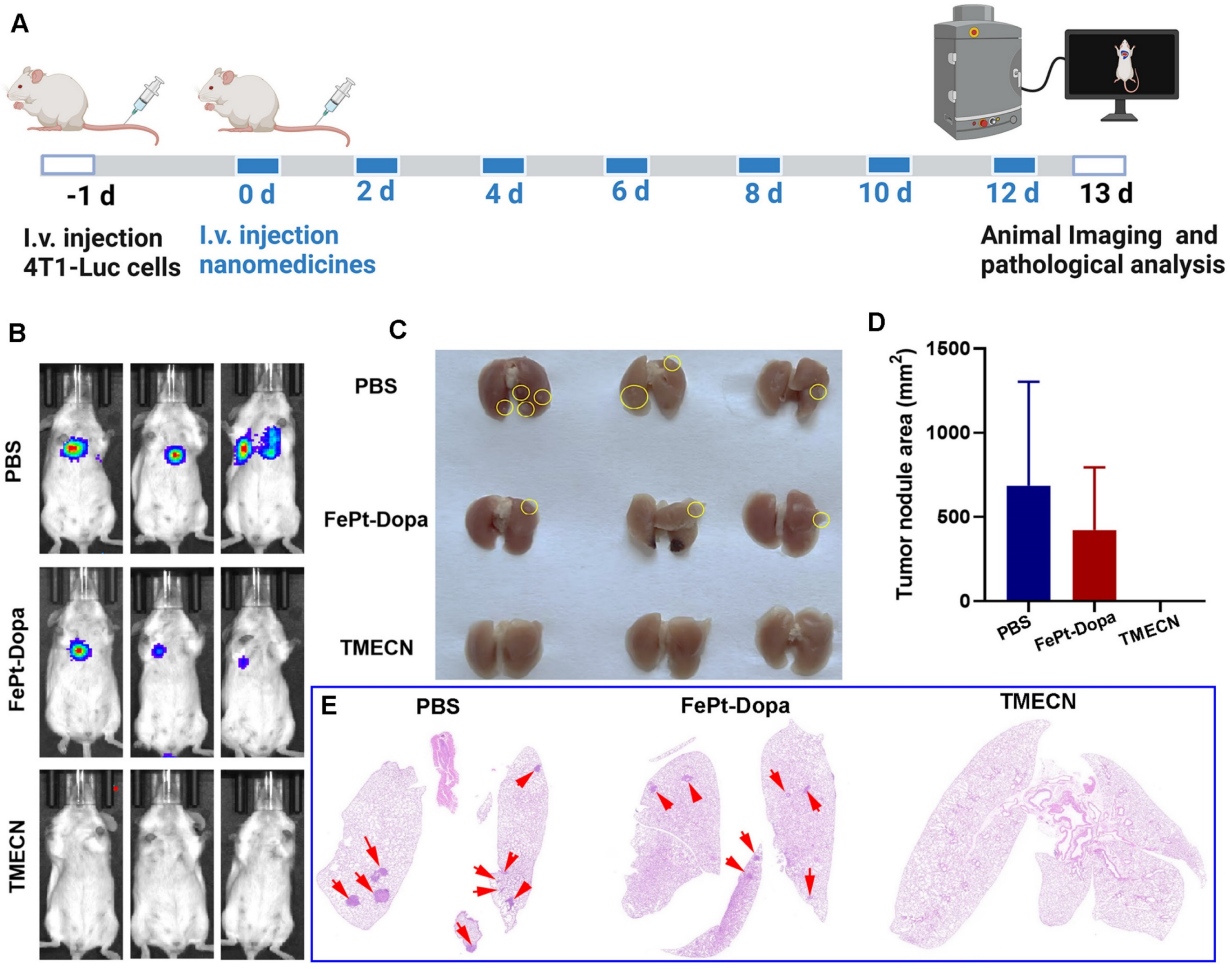
(A) Schematic diagram of construction and treatment of lung metastasis model. (B) BLI images of mice in each group. (C) Representative digital photos of anatomical lung tissue, metastatic tumor nodules marked with yellow circles. (D) Statistical results for the relative percentage of lung metastatic foci area from H&E staining images. (E) H&E staining of dissected lungs in each group. Metastatic tumor nodules marked with red arrow.

Encouraged by the immunostimulatory potency, TMECN is promising for the treatment of advanced metastatic cancers. Based on this, we further established a 4T1-luciferase (4T1-Luc) breast cancer metastasis model to investigate whether it could prevent tumor metastasis. The mice were intravenously injected with 4T1-Luc cells and treated with TMECN, FePt-Dopa or PBS for 7 times, respectively (**Figure 5A**). The tumor metastatic burdens were detected by bioluminescence imaging (BLI). The results exhibited that there was almost no bioluminescence in the lung tissues after TMECN treatment. The greater tumor burdens were observed in mice treated with FePt-Dopa, and the PBS group showed the strongest bioluminescence (**Figure 5B**). After treatment, the mice were executed and the lung tissues were excised. Many metastatic nodules were observed in the PBS group. Whereas, the lung tissues in the TMECN group were smooth and healthy, and almost no obvious tumor nodules were observed (**Figure 5C**). H&E staining results also showed that the lung tissue of mice treated with PBS and FePt-Dopa showed a large number of pulmonary metastatic nodules, however few significant pulmonary metastatic burdens were observed in the TMECN group (**Figure 5D-E**). These findings suggest that TMECN has great capability for inhibiting tumor lung metastasis.

### Aggregation-induced MRI contrast enhancement of TMECN* in vivo*

**Figure 6:**
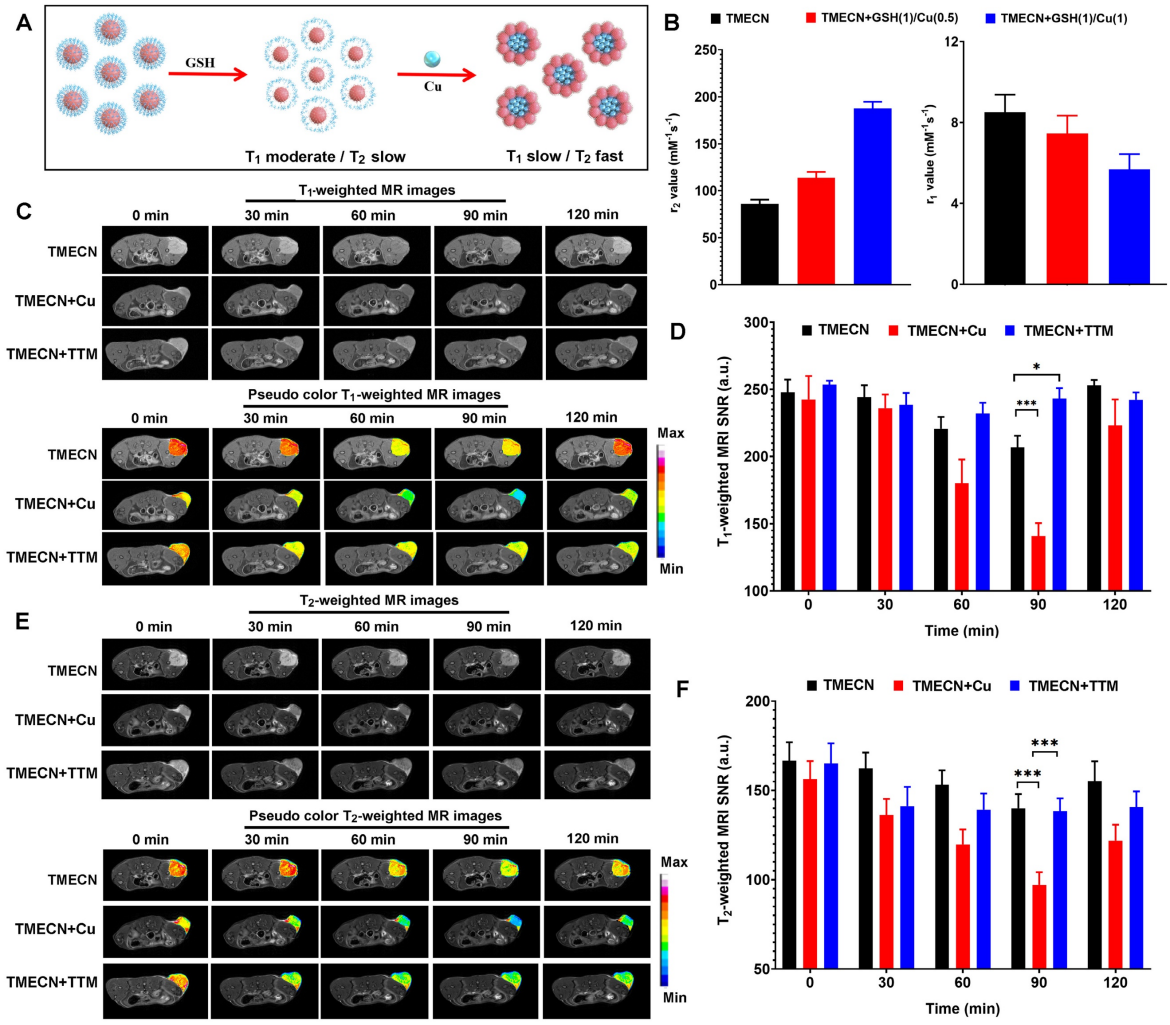
(A) Schematic diagram of aggregation-induced T_2_ contrast enhancement. (B) r_1_ and r_2_ values of TMECN treated with different concentrations of free Cu ions at the presence of GSH. (C) T1WI and (D) corresponding T_1_-weighted MRI signal to noise ratio of tumor in mice treated with TMECN, TMECN+Cu, and TMECN+TTM. (E) T_2_WI and (F) corresponding T_2_-weighted MRI signal to noise ratio of tumor in mice treated with TMECN, TMECN+Cu, and TMECN+TTM.

Upon entering cancer cells, monodispersed TMECN reacted with excessive GSH, exposed active groups, and then captured cellular copper ions, which self-assembled into magnetic nanocluster. Based on the mechanism of aggregation-induced MRI enhancement, magnetic nanocluster had stronger T_2_ MRI contrast performance and weaker T_1_ MRI contrast performance than monodispersed TMECN (**Figure 6A**). In order to verify this process, TMECN solution was incubated with 1 μg/mL of GSH and different concentrations of free Cu ions (0.5 μg/mL and 1.0 μg/mL) in turn, and then the transverse and longitudinal relaxation rates of TMECN were measured using an MRI scanner before and after treatment. As shown in **Figure 6B**, r_2_ value of TMECN significantly increased from 85.9±4.5 mM^-1^s^-1^ to 113.6±6.7 mM^-1^s^-1^, further following 187.5±7.2 mM^-1^s^-1^ after GSH and Cu ions treatment. Inversely, r_1_ value of TMECN gradually decreased. These results demonstrated that the presence of GSH and Cu^2+^ ions synergistically induced T_1_ to T_2_ switch MRI contrast of TMECN, which promoted more accurate diagnosis of tumor.

Encouraged by excellent switchable MRI contrast performance, we further investigated tumor MRI diagnostic ability of TMECN *in vivo*. 4T1 tumor-bearing mice were intravenously administrated with TMECN, TMECN+Cu, and TMECN+TTM at the dosage of 2 mg/kg, and then were observed using a high-field MRI device (7.0 T) at different time points. As shown in **Figure 6C**, T_1_-weighted images (T_1_WI) of tumor treated with TMECN gradually darkened with the increase of time, and the darkest image appeared at post-injection 90 min, following that the tumor began to brighten. The similar tendency was also observed in TMECN+Cu treated tumor. However, T_1_WI of tumor treated with TMECN+TTM had no significant variation with the increase of time, indicating that the clearance of Cu ions cannot induce self-assembly of TMECN and activate MRI contrast enhancement. In addition, the T_1_ signal noise ratio (SNR) of tumor was also collected using a MRIcro software (**Figure 6D**). The results indicated that the SNR of tumor treated with TMECN slightly decreased from 240.7±9.7 to 206.7±8.7, however TMECN+Cu dramatically decreased from 242.5±17.5 to 140.7±9.8, showing a Cu ion-accelerated T_1_ to T_2_ switchable MRI contrast effect. It was noted that the use of TTM significantly inhibited the SNR decrease of tumor, further confirming Cu ions-mediated T_1_-weighted MRI contrast enhancement. Next, we also explored T_2_-weighted image (T_2_WI) of tumor before and after different treatments. The similar results were also observed in T_2_WI. As shown in **Figure 6E**, T_2_WI of tumor in TMECN and TMECN+TTM groups slightly darkened at post-injection 90 min, however the T_2_WI of TMECN+Cu significantly darkened. Moreover, the darkest MR image appeared at post-injection 90 min. The T_2_ SNR of tumor in TMECN+Cu group sharply decreased from 156.3±10.2 to 97.1±7.1, and the corresponding T_2_ SNR change (∆SNR) reached up to be 37.7% (**Figure 6F**). Comparatively, the T_2_ ∆SNR of tumor in TMECN and TMECN+TTM were only 16.9% and 16.2%, respectively. These results indicated that Cu ions mediated self-assembly effect of TMECN significantly amplified T_1_ and T_2_ ∆SNR of tumor, contributing to accurate diagnosis of early tumor.

### Biosafety evaluation of TMECN *in vitro* and* in vivo.*

**Figure 7:**
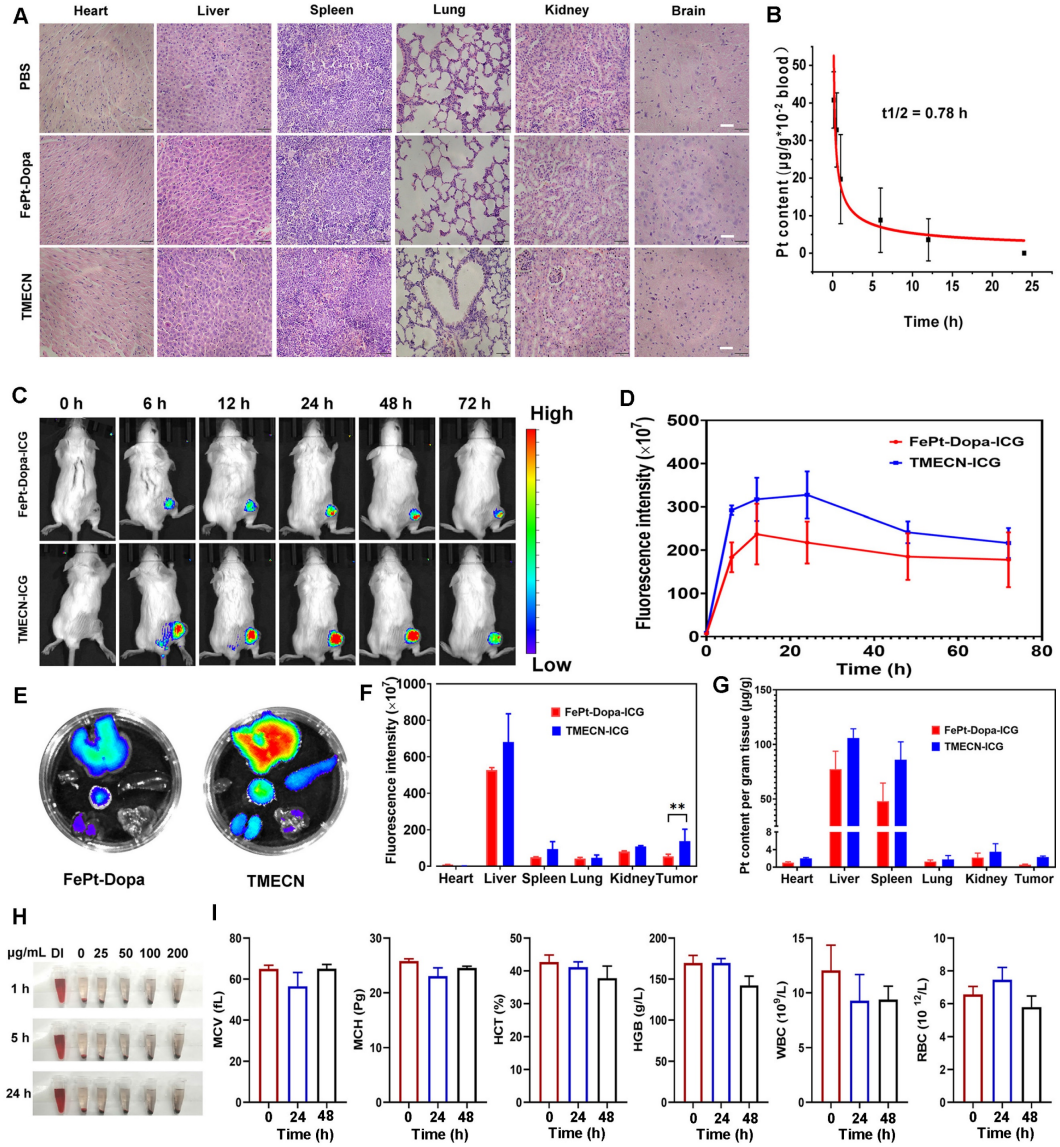
(A) H&E staining images of vital organs from the mice with different treatments. (B) Pharmacokinetics of TMECN in mice. (C) The fluorescence images and (D) corresponding fluorescence intensity of 4T1 tumor-bearing mice treated with FePt-Dopa-ICG and TMECN-ICG. (E) The fluorescence images and (F) corresponding fluorescence intensity of FePt-Dopa-ICG and TMECN-ICG in vital organs. (G) ICP-OES analysis for Pt content in 4T1 tumor and vital organs treated with FePt-Dopa and TMECN. (H) Hemolysis assay of TMECN at different concentrations. (I) Routine blood analysis data of the mice before and after TMECN treatments.

The biosafety of nanomaterials is prime importance for biomedical applications. Therefore, in order to confirm the damage of TMECN to normal tissues, the primary organs were collected and subjected to H&E staining for pathological analysis after the treatments. **Figure 7A** showed that there were no morphological changes in the heart, liver, spleen, lung, kidney, and brain tissues after TMECN treatment, demonstrating excellent tissue biosafety. In addition, the pharmacokinetic behavior of TMECN in the blood circulation was also examined via ICP-OES (**Figure 7B**). The blood half-life of TMECN was determined to be 0.78 h, and it could be absolutely cleared from the blood at 24 h post-injection.

In order to detect the enrichment of FePt-Dopa and TMECN at the tumor site, 4T1-bearing mice were intravenously injected with ICG labeled FePt-Dopa and TMECN at the dosage of 10 mg/kg, respectively. The biodistribution of nanoparticles was observed at different time points using a small animal imaging device. As shown in **Figure 7C-D**, the maximal accumulation of TMECN and FePt-Dopa in tumor site appeared at post-injection 48 h, moreover the accumulation of TMECN in tumor site was significantly higher than that of FePt-Dopa, demonstrating that TMECN easily accumulated in tumor and promoted the removal of Cu^2+^ ions. At post-injection 72 h, the mice were sacrificed. The vital organs were excised and imaged using a small animal imaging device. Meanwhile, the vital organs were also nitrated, and the Pt contents in organs were measured by ICP-OES. Results showed that FePt-Dopa and TMECN mainly distributed in liver and spleen, indicating that the TMECN was excreted out from body via hepatic pathway (**Figure 7E-G**). Moreover, Pt concentration of tumor treated with TMECN was higher than that with FePt-Dopa, demonstrating the excellent tumor targeting ability of TMECN* in vivo*. The hemolysis of TMECN was also evaluated, as revealed by the results (**Figure 7H** and **Figure S10A**), there was no significant damage to erythrocytes when TMECN concentrations ranged from 0 to 200 μg/mL. In addition, the blood routine examination and biochemical indexes including mean corpuscular volume (MCV), mean corpuscular hemoglobin (MCH), hematocrit (HCT), hemoglobin (HGB), white blood cell (WBC), red blood cells (RBC), alanine aminotransferase (ALT), aspartate aminotransferase (AST) and urea were determined. As shown in **Figure 7I** and **Figure S10B**, there had no significant variations before and after TMECN treatment for 24 h or 48 h. These results demonstrated that TMECN didn't damage liver function and renal function and showed excellent blood biocompatibility. In conclusion, the above analyses showed that the TMECN had excellent tissue biosafety and blood biocompatibility, showing the potential clinical application in cancer therapy.

## Conclusion

In summary, we successfully prepared a TME-responsive copper nanodeprivator for enhancing antitumor immunity and anti-angiogenesis. This copper nanodeprivator could inhibit tumor angiogenesis and reduce PD-L1 expression specifically in tumors, blocking cancer immune escape. *In vitro* results demonstrated that copper nanodeprivator could significantly inhibit the invasion and migration of tumor cells and PD-L1 expression. *In vivo* results further suggested that TMECN could effectively suppresses tumor growth and elicit antitumor immune responses. Furthermore, monodispersed TMECN quickly self-assemblied into TMECN nanocluster, which significantly strengthened MRI contrast difference of tumor and normal tissue and improved MRI diagnosis of tumor. In addition, TMECN significantly inhibited tumor relapse and metastasis with negligible systemic toxicity. Taken together, this study provides a novel theranostic approach through removing Cu in tumor to decrease PD-L1 expression, laying the foundation for clinical trials to evaluate copper nanodeprivator as immune checkpoint inhibitors.

## Supplementary Materials

Supplementary materials and methods and figures.
