# Supplementary material for: MRI guided copper deprivator activated immune responses and suppressed angiogenesis for enhanced antitumor immunotherapy
Source: Theranostics. 2025 Jan 1;15(2):546–59. doi: 10.7150/thno.102556 (PMC11671392; doi:10.7150/thno.102556)
Supplement: Supplementary file 1 — Supplementary materials and methods and figures. [file thnov15p0546s1.pdf]

MRI guided copper deprivator activated immune responses and suppressed angiogenesis for enhanced antitumor immunotherapy

Yinfeng Wang[1,#], Peng Wang[1,#], Huimin Li[1,#], Miao Yan[1], Feixue Ni[1], Li Zhang[3,*], Zhen Zhao[2,*], Wenjuan Gao[1,*], and Guilong Zhang[1,*]

[1] School of Pharmacy, Shandong Technology Innovation Center of Molecular Targeting and Intelligent Diagnosis and Treatment, Binzhou Medical University, Yantai 264003, P.R. China

[2] Department of Vascular Surgery, Shanghai Ninth People's Hospital, Shanghai Jiaotong University School of Medicine, Shanghai, China

[3] Department of Urology, the First Affiliated Hospital of Anhui Medical University, Institute of Urology, Anhui Medical University and Anhui Province Key Laboratory of Genitourinary Diseases, Anhui Medical University, Hefei 230022, Anhui, People's Republic of China

* **Corresponding author:** Li Zhang, Ph.D, e-mail: lzhang@ahmu.edu.cn; Zhen Zhao, Ph.D., e-mail: zhenzhaolmu@163.com; Wenjuan Gao, Ph.D., e-mail: gaowj2020@bzmc.edu.cn; Guilong Zhang, Ph.D., e-mail: glzhang@bzmc.edu.cn

[#] These authors contributed equally to this work.

Materials and methods

Materials: Platinum Bis ($C_{10}H_{14}O_4Pt$), 1,2-Hexadecanediol ($C_{16}H_{34}O_2$), Dialysis Membranes, and aminopolyethylene glycol sulfhydryl (NH_2-PEG-SH) were acquired from Shanghai Yuanye Bio-Technology Ltd (China). Nonacarbonyldiiron ($C_9Fe_2O_9$), Dibenzyl ether ($C_{14}H_{14}O$), Oleic acid, Oleylamine, meso-2,3-Dimercaptosuccinic acid (DMSA), and 2,2'-bipyridine-4,4'-dicarboxylic acid (Bpy-COOH) were obtained from Aladdin Reagents Ltd (China). TNF-α, IFN-γ and IL-6 Elisa Kit were acquired from Shanghai Yuanke Biotechnology (China). Antibodies against EGFR, p-EGFR, STAT3, p-STAT3 were acquired from Santa Cruz Biotechnology (USA). CRT, PD-L1 antibodies were obtained from Cell Signaling Technology (USA). ECL chemiluminescence kit was obtained from Biosharp.

Synthesis of FePt: Typically, FePt nanoparticles are prepared by high temperature thermal degradation. $Pt(acac)_2$ (0.1001 g), 1,2-hexadecanediol (0.2001 g) and diphenyl ether (20 mL) were mixed in a three-neck flask under nitrogen. The mixed solution was heated to 100 °C and oleic acid (160 μL) and oleylamine (170 μL) were added rapidly. Oleic acid (500 μL) and oleic acid (500 μL) were added for another 30 min. After waiting for 13 min, $Fe_2(CO)_9$ (0.3657 g) was added immediately, condensed, refluxed at 291.2 °C for 3 h, and cooled to room temperature. The reaction solution was washed three times with ethanol and hexane and centrifuged to obtain the black product.

Synthesis of FePt@DMSA: FePt (10 mg) was redissolved in toluene (20 mL), next DMSA (90 mg) and DMSO (5 mL) were added into the solution. The mixture was incubated for 48 h at room temperature under stirring. Then, the obtained products were separated by centrifugation and washed by ethanol at least three times. After that, FePt-DMSA was redispersed in the distilled water and then pH of the solution was adjusted to 10 using sodium hydroxide. Afterwards, the FePt-DMSA solution was filtrated using 100 nm filter membrane and then the solution was adjusted to neutral condition.

Synthesis of TMECN: TMECN was synthesized by a two-step thiol-disulfide exchange reaction. BPy-COOH (100 mg) and NH_2-PEG-SH (100 mg) were added

into DMSO solution (20 mL) under magnetic stirring. Subsequently, 1-(3-Dimethylaminopropyl)-3-ethylcarbodiimide (EDC, 100 mg) and N-hydroxysuccinimide (NHS, 100 mg) were further added into the resulting solution and continuously stirred overnight. After that, the DMSO solution of FePt@DMSA (5 mg/mL, 20 mL) was mixed into the resulting solution, meanwhile H_2O_2 (1 mL) was also added. The suspension was stirred for 24 h at room temperature in the dark. At the end of the reaction, the product was separated by centrifugation and washed three times with water.

Evaluation of copper chelating capacity of TMECN: First, the copper removal efficiency of TMECN was determined by ICP-MS. In general, different concentrations of TMECN, copper sulfate (10 μg/mL), and different concentrations of GSH were mixed, stirred for 6 h, and the mixture was centrifuged and filtered. The filtrate was analyzed for copper concentration by ICP-MS and the copper removal efficiency was calculated accordingly.

Cellular Uptake: First, 4T1 cells were inoculated in confocal dishes (20,000 cells/well in 2 mL 1640 medium) and cultured for 24 h. Cells were incubated with TMECN at different concentrations (10, 20 and 40 μg/mL) for 6 h. Then, cells were incubated with Hoechst 33342 (10 μg/mL) for 20 min in the dark and then repeatedly washing. Finally, the cells were observed by fluorescence microscopy. To quantify the time- and concentration-dependent internalization behavior, 4T1 cells were treated with the same concentration (40 μg/mL) for 1, 3, and 6 h and with different concentrations (10, 20, and 40 μg/mL) for 6 h. Then, repeated washes were performed. Finally, the Pt concentration in the cells was analyzed by ICP-MS.

Cytotoxicity assay: Cells (4T1, PC3, C166, L929) were inoculated in 96-well plates and then treated with different concentrations of TMECN (0, 1, 5, 10, 20, 40 μg/mL) for 24 h. MTT reagent was added to each well and incubated for a further 4 h. Supernatant was discarded, and 150 μL of dimethylsulfoxide (DMSO) was added to each well to dissolve the methazan crystals. After shaking for 30 s, the absorbance at 490 nm was recorded with a microplate reader and the percentage of residual cell viability was calculated.

4T1 cells were pretreated with L-buthionine sulfoximine (BSO) (100 μM) for 8 h, and then re-incubated with TMECN (40 μg/mL), TMECN (40 μg/mL) + GSH (5 mM) solution for 24 h, respectively. Cell viability was then calculated as before.

In vitro cytokine measurement: 4T1 cells were plated at a density of 2×10^5 cells in 6 well plate and incubated for 24 h. Cells were then treated with PBS, FePt-Dopa (40 μg/mL) or TMECN (40 μg/mL) for 48 h. Collect the medium in a sterile centrifuge tube and centrifuge for about 20 min (2500 rpm). Detect the release of INF-γ, IL-6 and TNF-α from the medium using the ELISA Kit according to the manufacturer's instruction.

Western Blot Analysis: 4T1 cells were incubated with PBS, FePt-Dopa (40 μg/mL) or TMECN (40 μg/mL) for 24 h, respectively. Then, the cells were harvested with lysis buffer (50 mM Tris-HCl [pH 7.4], 150 mM NaCl, 0.1% SDS, 1% NP-40, 0.5% sodium deoxycholate and pre-mixed with protease inhibitor cocktail (B14001, Biomake). BCA Protein Assay Kit (Beyotime Biotechnology) was conducted to detect the concentration of proteins. Then, the proteins were separated by sodium dodecyl sulfate-polyacrylamide gel electrophoresis (SDS-PAGE) and moved to a polyvinylidene fluoride (PVDF) membrane. The membranes were blocked by 5% skim milk. The bands were detected using the following primary antibodies: anti-p-EGFR, EGFR, p-STAT3, STAT3, p-AKT, AKT, PD-L1, CTR and GAPDH overnight at 4 °C. The membranes were incubated with appropriate horseradish peroxidase (HRP)-conjugated secondary antibodies for 1 h at room temperature (RT). Finally, the specific protein bands were captured with an enhanced ECL chemiluminescence kit.

Transwell assay: 4T1 and PC3 cells were seeded into the upper chambers pre-coated with Matrigel matrix at a density of 5×10^4 cells/well and then cultured in serum-free media containing PBS, free FePt-Dopa (40 μg/mL), TMECN (40 μg/mL), TMECN (40 μg/mL) + GSH (5 mM) and TMECN (40 μg/mL) + Cu (10 μg/mL) + GSH (5 mM), respectively. Then, the cells were allowed to migrate for 24 h toward the lower chambers filled with culture media containing 10% FBS. Next, the cells that migrated to the bottom side of the membranes were stained with 1% crystal violet.

Wound-healing assay: 4T1 and PC3 cells were seeded into 60-mm dishes at a density of 8×10^5 cells/well for 24 h to grow a monolayer. Next, a linear wound was created across the middle of the well surface using a pipette tip, and the cells were then incubated in serum-free media containing PBS, FePt-Dopamine (20 μg/mL), TMECN (20 μg/mL), TMECN (20 μg/mL) + GSH (5 mM) and TMECN (20 μg/mL) + Cu (10 μg/mL) + GSH (5 mM). At 24 h, the wound widths from 3 defined field under an inverted microscope were captured.

Tube-formation assay: C166 cells were incubated with PBS, FePt-Dopa (40 μg/mL), TMECN (40 μg/mL), TMECN (40 μg/mL) + GSH (5 mM) and TMECN (40 μg/mL) + Cu (10 μg/mL) + GSH (5 mM) for 24 h, respectively. Afterwards, Matrigel matrix were added to the 96-well plates and then incubated successively on the ice for 10 min and at 37 ℃ for 1 h to allow solidification. Next, C166 cells in 50 μL serum-free DMEM containing 8×10^4 cells were seeded onto these matrices and further incubated for 3 h. Finally, 3 random fields in each well were imaged using a computer-based phase-contrast microscope and the endothelial cell tubes were subsequently counted.

Anticancer activity assessment in vivo: BALB/c female mice (four weeks old) were purchased from the GemPharmatech Biotechnology Co., LTD (Nanjing, China). All animal experiments were approved by the Research Institute Ethics Committee of Binzhou Medical University and were conducted according to the guidelines on the use and care of laboratory animals of Binzhou Medical University. 4T1 tumor model was established by subcutaneous injection of 4T1 (1×10^5) cells into the right hind legs of BALB/c mice. When the tumor volume is about 60 mm^3, the in vivo experiments were carried out. The tumor-bearing mice were divided into three groups (n = 4): (a) PBS; (b) FePt-Dopa; (c) TMECN. The mice were intravenously injected with these drugs. Subsequently, the tumor volume and mice weight were recorded at every day intervals. The mice were sacrificed on day 15 and major organs and tumor tissues were excised for further histological analysis.

H&E and IF Staining: Tumor tissues from 4T1 tumor-bearing mice were fixed in 4% paraformaldehyde, dehydrated, paraffin-embedded, sectioned 5 mm thick, and

then deparaffinized. The slices were stained by hematoxylin and eosin (H&E). Then, the slices were dehydrated and mounted with neutral balsam. For immunofluorescence (IF) staining, the slices were blocked with 5% bovine serum albumin (BSA), incubated with primary antibodies (VEGF-A, CD31, CRT, HMGB1 and PD-L1) overnight at 4 °C. Subsequently, the sections were stained with proper secondary antibodies conjugated with Alexa Fluor 488 or Alexa Fluor 594 for 1 h at RT. Finally, cell nuclei were stained with DAPI. These images were photographed by a fluorescence microscope (Axio observer 5, Zeiss, Germany).

In vivo copper stripping triggers anti-tumor immunotherapy: To evaluate the therapeutic effect of copper stripping-activated immunotherapy, mice with bilateral tumors were randomly divided into PBS, FePt-Dopa, TMECN (n = 3). NPs were intravenously injected every two days (Pt concentration: 2 mg/kg, 100 μL) were injected intravenously every two days for seven treatments. The distal tumors were then inoculated, and mice were executed on day 27. Primary tumors were excised, and single-cell suspensions were prepared and stained with anti-CD45 Ab-FITC, anti-CD3 Ab-APC, anti-CD4 Ab-PE, anti-CD8 Ab-PerCP-Cy5.5 for analysis of tumor-infiltrating lymphocytes. The infiltration of NK cells was marked by CD45-FITC, CD3-APC and CD49b-PE antibodies. The DC maturation was stained with CD11c-BV421, CD80-FITC and CD86-APC antibodies. The weights of distant tumors in mice were also recorded.

Copper stripping mediated MRI enhancement: All MRI experiments were performed using a 7.0 T MRI scanner. T_1 and T_2 value of nanoparticles were measured via a series of inversion-prepared fast spin-echo sequence and a multiecho spin echo (MESE) sequence, respectively. For T_1 measurement, the parameters were as follows: TR/TE = 150, 300, 600, 1000, 2000, 4000, 8000/13 ms; FOV = 210 × 210 mm; Matrix size = 232 × 232; slice thickness = 3.0 mm (5 slices, gap=0). For T_2 measurement, the parameters were as follows: TR/TE = 5000/10, 20, 30, 40, 50, 60, 70, 80, 90, 100 ms; FOV = 210 × 210 mm; Matrix size = 232 × 232; slice thickness = 3.0 mm (5 slices, gap=0). For in vivo MRI experiment, T_1-weighted image (T_1WI) and T_2-weighted image (T_2WI) of tumor in mice were acquired at the axial planes at

different time points. For T_1WI, the parameters were as follows: TR/TE = 1,500/7 ms, slice thickness = 1 mm, slice spacing = 0 mm, FOV = 40 × 40 mm^2, matrix = 256 × 256. For T_2WI, the parameters were as follows: TR/TE = 5,000/40 ms, slice thickness = 1 mm, slice spacing = 0 mm, FOV = 40 × 40 mm^2, matrix = 256 × 256. For T_1 map imaging, the parameters were as follows: TR/TE = 5,000/8 ms, slice thickness = 1 mm, slice spacing = 0 mm, FOV = 40 × 40 mm^2, matrix = 256 × 256. For T_2 map imaging, the parameters were as follows: TR/TE = 5,000/9 ms, slice thickness = 1 mm, slice spacing = 0 mm, FOV = 40 × 40 mm^2, matrix = 256 × 256.

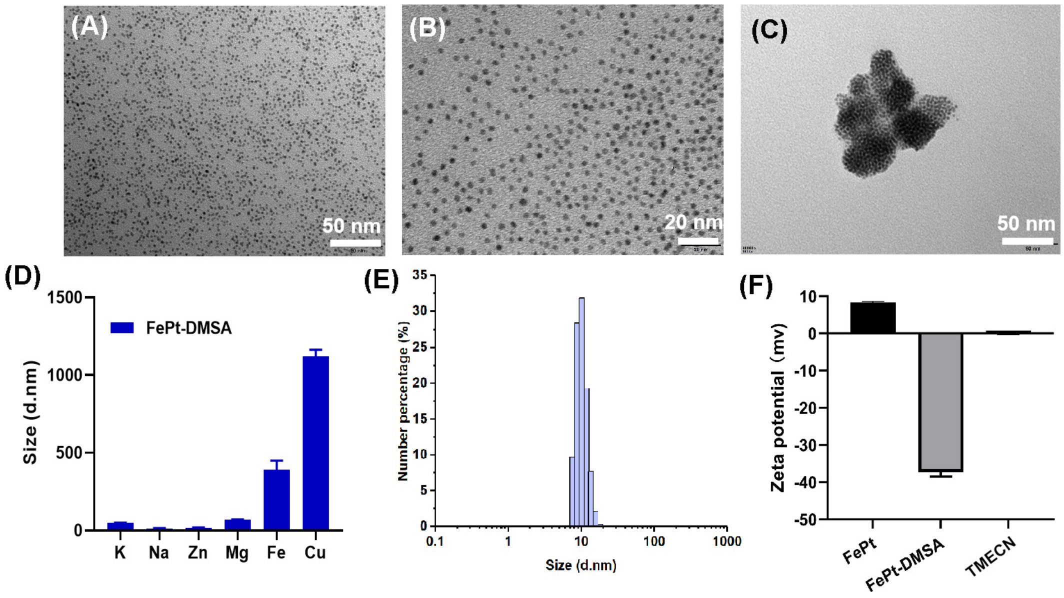

Figure S1. TEM observation of (A) hydrophobic FePt nanoalloy, (B) FePt-DMSA, and (C) aggregates after chelating copper with FePt-DMSA nanoparticles. (D) Hydrodynamic particle size change of FePt-DMSA at the presence of different metal ions. (E) Hydrodynamic size of TMECN. (F) Zeta potential of FePt, FePt-DMSA, and TMECN.

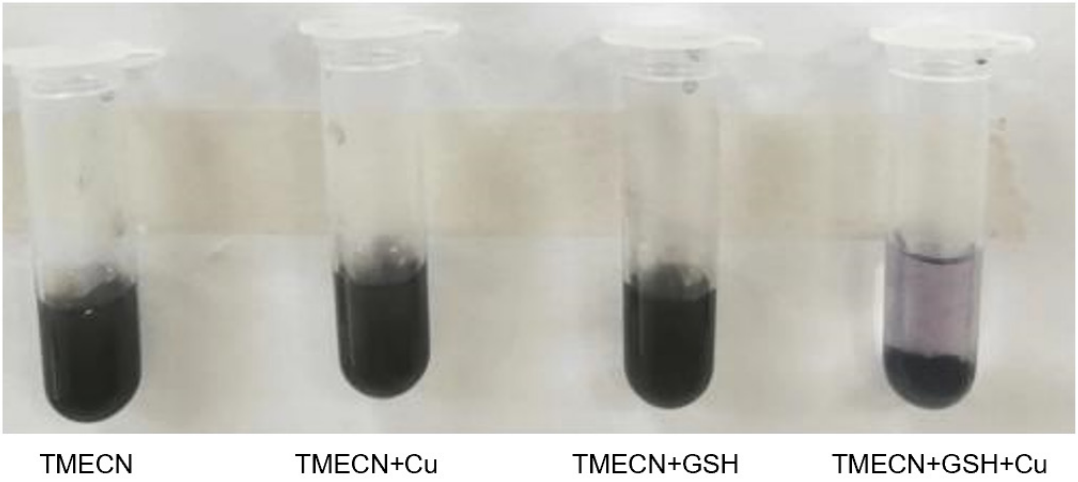

Figure S2. Photographs of TMECN incubated with free Cu^{2+} (60 μM), GSH (5 mM), and GSH (5 mM) + Cu^{2+} (60 μM) for 30 min.

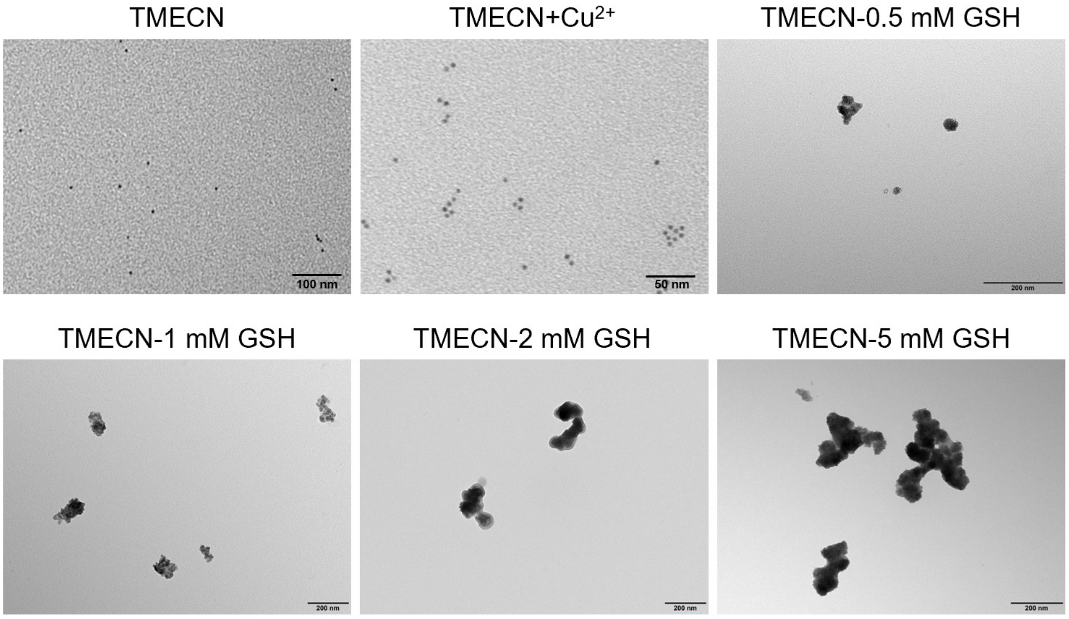

Figure S3. TEM images of TMECN and TMECN-Cu after treatment with different concentrations of GSH.

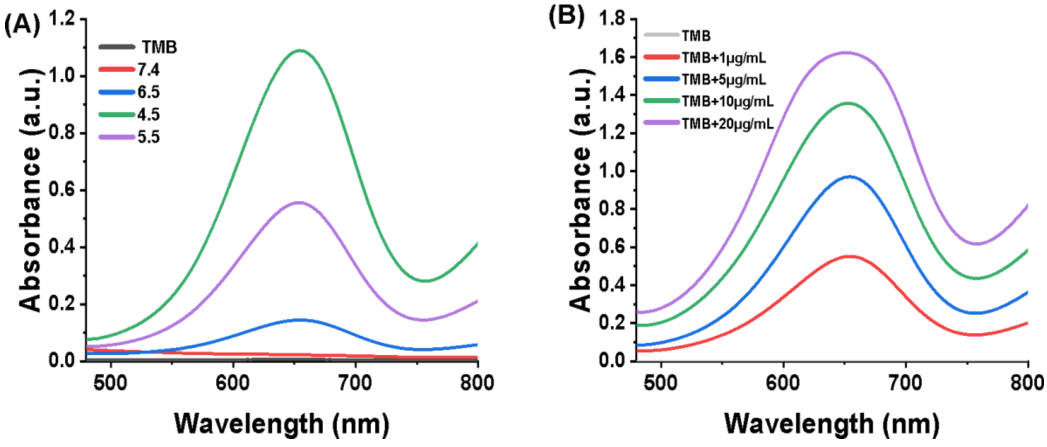

Figure S4. (A) UV-Vis spectra analysis for TMECN-catalyzed H_2O_2 produced ·OH in the different pH of TMB solutions. (B) UV-Vis spectra analysis for different concentrations of TMECN-catalyzed H_2O_2 produced ·OH in the TMB solutions (pH 4.5)

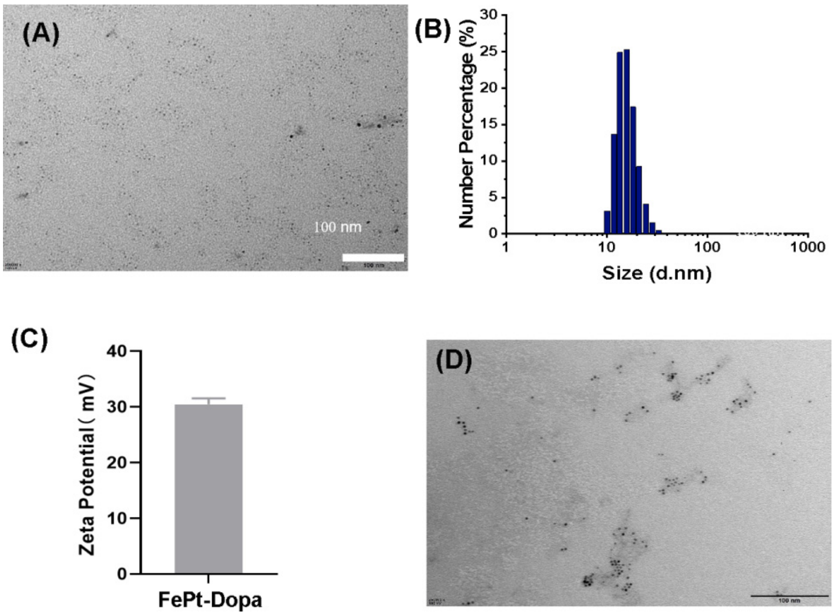

Figure S5. (A) TEM image of FePt-Dopa. (B) Hydrodynamic size of FePt-Dopa. (C) Zeta Potential of FePt-Dopa. (D) TEM image of FePt-Dopa after free Cu^{2+} treatment.

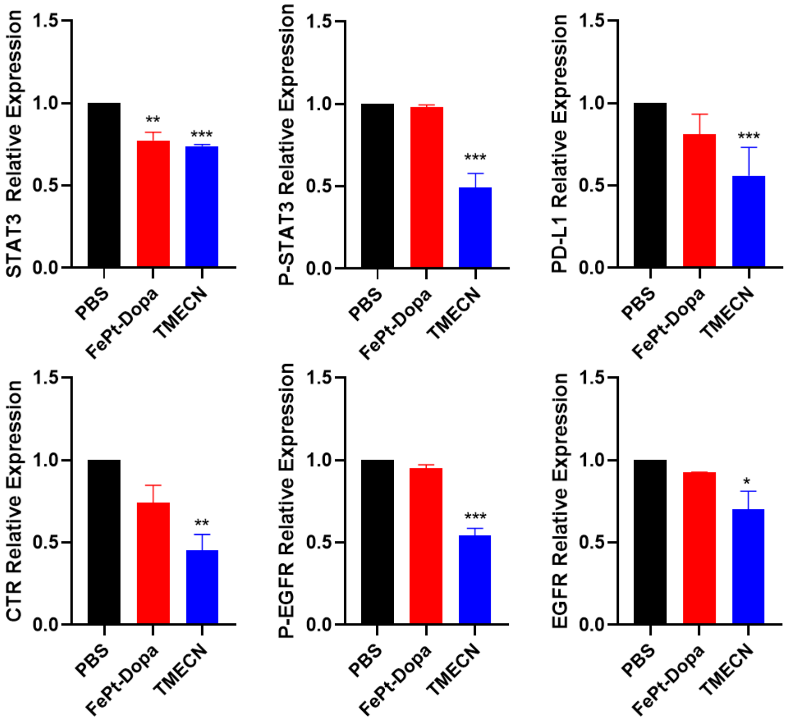

Figure S6. Quantifications of the related protein expression in 4T1 cells treated with PBS, FePt-Dopa, and TMECN.

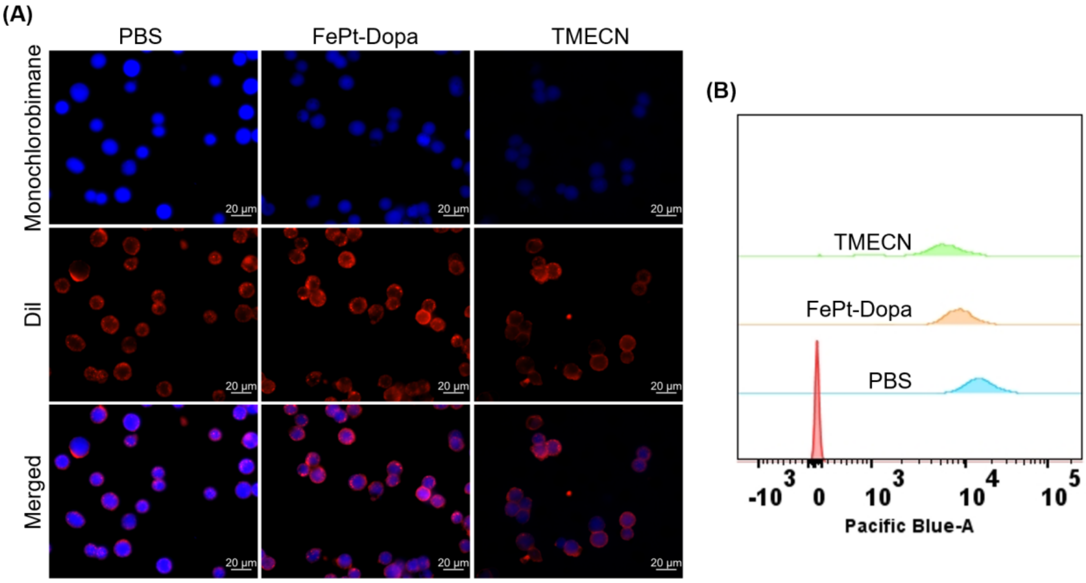

Figure S7. (A) CLSM observation and (B) flow cytometry analysis for GSH level in 4T1 cells treated with different samples.

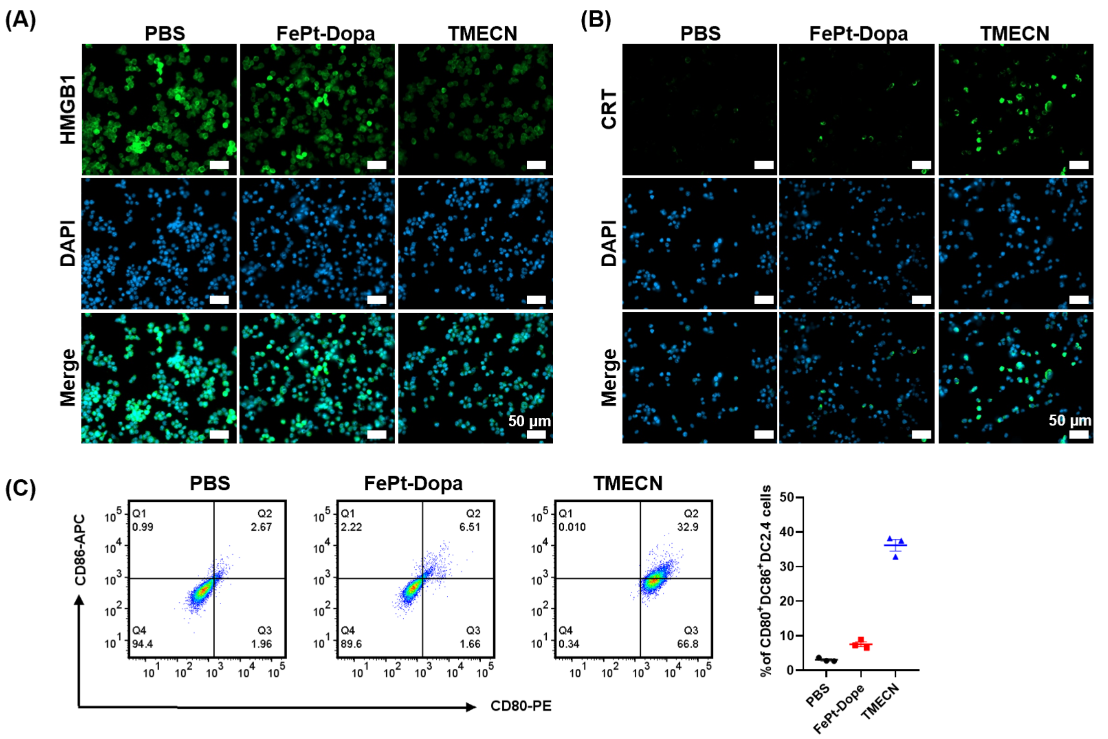

Figure S8. Immunofluorescence images of HMGB1 (A) and CRT (B) on 4T1 cells receiving different treatments for 24 h. (C) The proportion of $CD80^{+}CD86^{+}$ DCs evaluated by flow cytometry after different treatments.

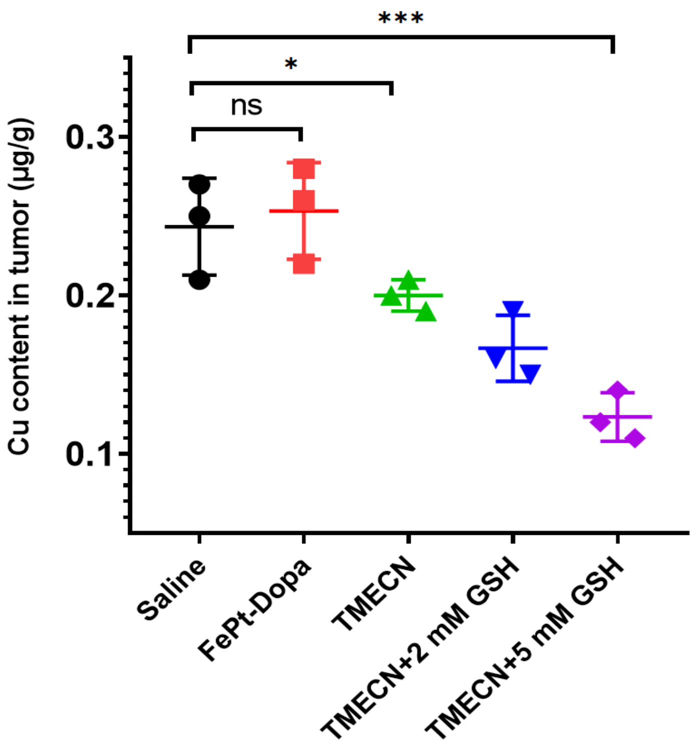

Figure S9. Copper levels of tumor after different treatments.

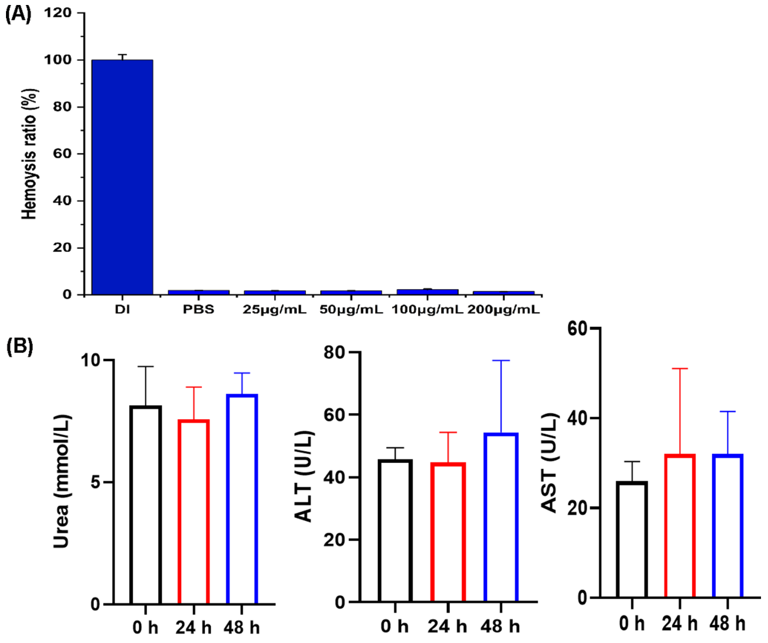

Figure S10. (A) UV-Vis absorption spectra of erythrocyte supernatants treated with TMECN. (B) The blood biochemical indexes of the mice before and after TMECN treatments.
